# MCQs in Psychiatry for Medical Students

**DOI:** 10.1192/pb.bp.116.054189

**Published:** 2017-06

**Authors:** Suzanne Dash

Love them or loathe them, most medical student written examinations now take the form of multiple choice questions (MCQs). Some medical educators dislike this assessment style, suggesting it encourages students to learn isolated facts in a superficial way. Yet, undeniably, MCQs provide an objective, time-efficient manner of evaluation.

*MCQs in Psychiatry for Medical Students* is a valuable resource for medical students undertaking their psychiatry rotations. It includes MCQs and extended matching items grouped into chapters concerned either with a type of disorder – for example, psychotic disorders and alcohol and substance misuse disorders – or another important aspect of psychiatry, such as physical health, pharmacological treatments, psychology and psychotherapy.

Each MCQ is accompanied by a paragraph or two explaining the correct answer. More information is provided than is strictly necessary to understand the answer, but this is illuminating rather than turgid. The 400-plus contemporary references encourage the reader to consider issues in more depth than the superficial learning style many associate with MCQs, making the scope of this book potentially greater than is obvious from its title. In contrast, the three extended matching item questions in each chapter are not followed by explanations, making them far less informative.

Writing good MCQ distractor items is a challenge, and in a few places – especially questions on risk factors and protective factors – it is possible to guess the answer by eliminating answers simply based on whether they describe something positive or negative.

This is a must-have title for all medical students; it will pique the interest of many students and may even assist in recruiting future psychiatrists to the profession.

